# Supplementary material for: High-Voltage Lithium-Ion Battery Using Substituted LiCoPO4: Electrochemical and Safety Performance of 1.2 Ah Pouch Cell
Source: Materials (Basel). 2020 Oct 7;13(19):4450. doi: 10.3390/ma13194450 (PMC7579286; doi:10.3390/ma13194450)
Supplement: Supplementary file 1 [file materials-13-04450-s001.pdf]

materials

*Supplementary Materials*

# High-Voltage Lithium-Ion Battery Using Substituted LiCoPO4: Electrochemical and Safety Performance of 1.2 Ah Pouch Cell

**Dongqiang Liu [1,*], Chisu Kim [1], Alexis Perea [1], Dubé Joël [1], Wen Zhu [1], Steve Collin-Martin [1], Amélie Forand [1], Martin Dontigny [1], Catherine Gagnon [1], Hendrix Demers [1], Samuel Delp [1], Jan Allen [1], Richard Jow [2] and Karim Zaghib [3,*]**

1 Center of Excellence in Transportation Electrification and Energy Storage (CETEES), Hydro Québec, 1806 Boulevard Lionel-Boulet, Varennes, QC J3X 1S1, Canada; kim.chisu@hydroquebec.com (C.K.); Perea.alexis2@hydroquebec.com (A.P.); dube.joel@hydroquebec.com (D.J.); Zhu.wen@hydroquebec.com (W.Z.); Collin-Martin.steve2@hydroquebec.com (S.C-.M.); forand.amelie@hydroquebec.com (A.F.); dontigny.martin@hydroquebec.com (M.D.); gagnon.catherine3@hydroquebec.com (C.G.); Demers.Hendrix@hydroquebec.com (H.D.); samuel.delp.ctr@mail.mil (S.D.); jan.l.allen8.civ@mail.mil (J.A.)
2 U.S. Army Research Laboratory, 2800 Powder Mill Road, Adelphi, 20783 MD, USA; t.r.jow.civ@mail.mil
3 Department of Mining and Materials Engineering, McGill University, 845 Sherbrooke Street West, Montréal, QC H3A 0G4, Canada
* Correspondence: liu.dongqiang@hydroquebec.com (D.L.); karim.zaghib@mcgill.ca (K.Z.)

Received: 4 September 2020; Accepted: 2 October 2020; Published: 7 October 2020

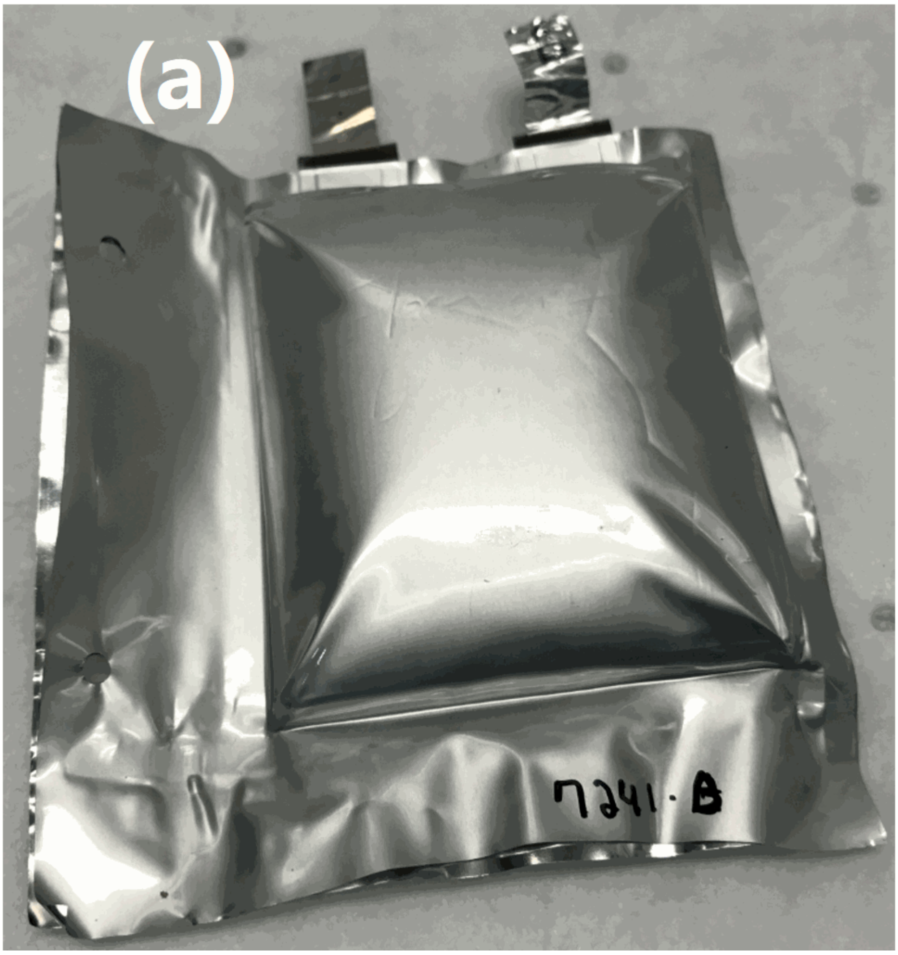

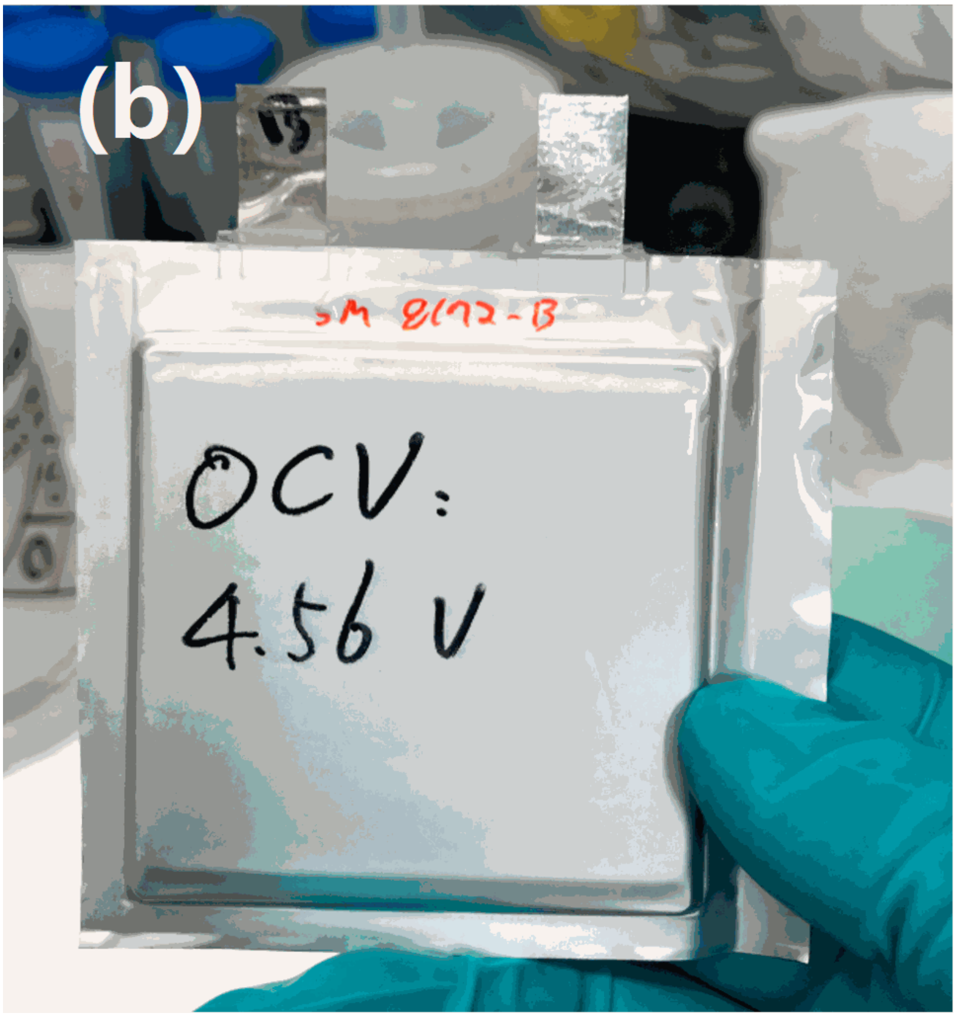


**Figure S1.** (**a**) 1.2 Ah LCP pouch cell using EC-EMC commercial electrolyte after cycling at 25 °C. (**b**) 1.2 Ah LCP pouch cell using Py13FSI ionic liquid electrolyte after cycling at 25 °C.

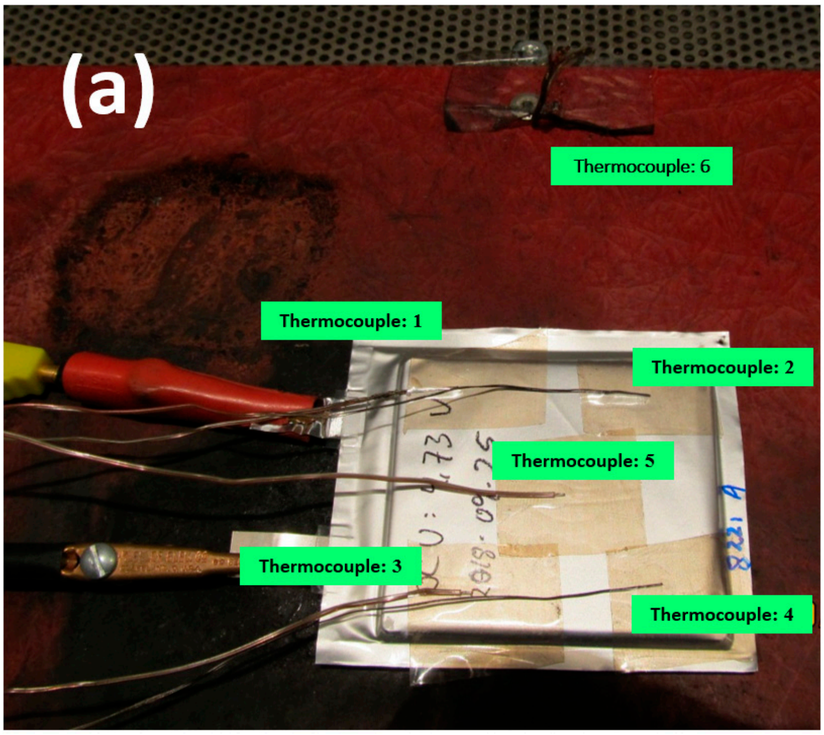

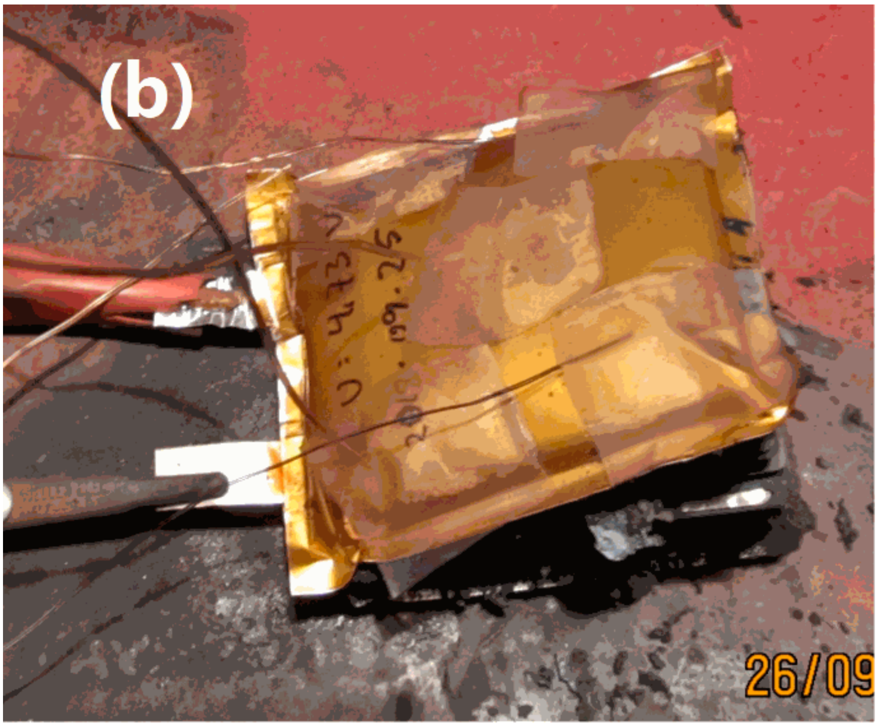


**Figure S2.** Image of the 1.2 Ah LCP pouch cell before (**a**) and after (**b**) the hot box test, number 1–6 in S2 (**a**) corresponding to the thermocouples in the test.

© 2020 by the authors. Licensee MDPI, Basel, Switzerland. This article is an open access article distributed under the terms and conditions of the Creative Commons Attribution (CC BY) license (http://creativecommons.org/licenses/by/4.0/).
